# Contextualizing the COVID-19 pandemic’s impact on food security in two small cities in Bangladesh

**DOI:** 10.1177/0956247820965156

**Published:** 2021-04

**Authors:** Hanna A Ruszczyk, M Feisal Rahman, Louise J Bracken, Sumaiya Sudha

**Keywords:** Bangladesh, COVID-19, food security, informal settlements, middle class, pandemic, secondary and small cities, urban

## Abstract

The COVID-19 pandemic is an evolving urban crisis. This research paper assesses impacts of the lockdown on food security and associated coping mechanisms in two small cities in Bangladesh (Mongla and Noapara) during March to May 2020. Due to restrictions during the prolonged lockdown, residents (in particular low-income groups) had limited access to livelihood opportunities and experienced significant or complete loss of income. This affected both the quantity and quality of food consumed. Coping strategies reported include curtailing consumption, relying on inexpensive starchy staples, increasing the share of total expenditure allocated to food, taking out loans and accessing relief. The pandemic has exacerbated the precariousness of existing food and nutrition security in these cities, although residents with guaranteed incomes and adequate savings did not suffer significantly during lockdown. While coping strategies and the importance of social capital are similar in small and large cities, food procurement and relationships with local governments show differences.

**Figure:**
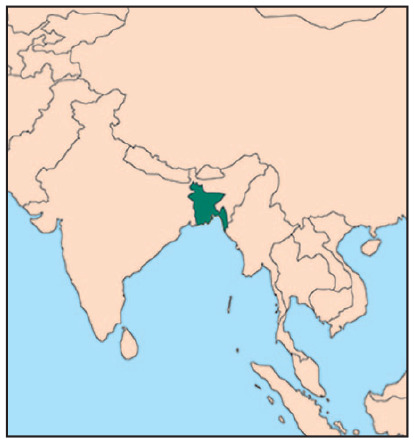


## I. Introduction

The United Nations declared on 20 May 2020 that the COVID-19 pandemic was more than a health emergency. The unfolding economic and social consequences of the pandemic are exposing high vulnerabilities and inequalities in food systems around the world and will have an extended impact. As economies shrink, employment and livelihood opportunities are expected to decline, diminishing the global poverty reduction gains of the last 30 years and forcing many families into food insecurity and starvation.^(1)^ COVID-19 will require coordinated responses to address the tremendous challenges emerging from its multifaceted impacts.^(2)^

Bangladesh reported its first case of COVID-19 on 7 March 2020, and the government of Bangladesh announced a *“general holiday with restrictions on movement”* effective from 26 March 2020. (This is referred to as the “lockdown” throughout this paper.) The national lockdown was eased at the beginning of June, although local restrictions remain in some parts of the country that have growing caseloads. As of 18 August 2020, Bangladesh had 282,344 cases of COVID-19 and 3,740 people had died from the virus.^(3)^ The Asian Development Bank, in a hypothetical worst-case scenario, predicted that Bangladesh could lose 1.1 per cent of GDP growth and 894,930 formal jobs due to the pandemic.^(4)^

While there are timely online academic repositories of policy advice and stock-taking assessments of local communities and their networks that are self-organizing to cope with the evolving crisis,^(5)^ these are largely focused on major cities and little is known about the lived experiences of residents in smaller, less prominent cities. Rapid response research conducted in April 2020 by the Power and Participation Research Centre and BRAC Institute of Governance and Development of Bangladesh^(6)^ observed a steep drop in income, extreme uncertainty of livelihoods and a contraction in consumption. Respondents from urban informal settlements and rural areas suffered an income drop of 75 per cent and 62 per cent respectively. The paper further reported that the income shock across all income groups led to a contraction in food consumption, as evidenced by a reduction in food expenditure of 28 per cent for urban informal settlement respondents and 22 per cent for rural respondents. However, it remains unclear how residents in smaller cities are coping with the food, social and economic disruptions associated with COVID-19.

This research paper addresses this knowledge gap on urban food (in)security during the COVID-19 pandemic in two small urbanizing cities in Bangladesh (Mongla and Noapara). The paper is based on two distinct periods of empirical work. The first round of fieldwork was conducted in September–October 2019 and explored subjective understanding of the liveability of regional cities from the perspectives of a range of residents and stakeholders. The second round of fieldwork, building on the first, was conducted in May–July 2020 and was a rapid analysis to understand the challenges imposed by COVID-19 on food (in)security and coping mechanisms during the nine-week lockdown period of March–May 2020.

## II. Small Cities, Urbanizing Bangladesh and Food Security

### a. Academically ignored small cities

The lack of attention given to smaller cities^(7)^ is a self-imposed limitation on our understanding of the urban throughout the global South. Following Hardoy and Satterthwaite’s^(8)^ seminal book on life in the urban world, urban scholars^(9)^ have recently argued for a renewed engagement and research into academically ignored secondary and small cities in the global South. Theoretical and methodological efforts have yet to position smaller and/or more regional secondary cities front and centre in research, and yet it is in these cities where most city dwellers reside.^(10)^ These residents include those categorized as poor, working class and lower-middle class (in such professions as mechanics and food market management)^(11)^; people from all groups aspire to a future in the city. These cities are also characterized by local governments with more curtailed capacities, minimal funding and resources, and often a lack of political power to fulfil their responsibilities.^(12)^

The relationship between food security, food systems and sustainability needs engaged consideration within these small cities.^(13)^ Understanding this relationship is crucial because urban poverty and food insecurity are interrelated. Our insights build on the important work initiated in *Environment and Urbanization*’s October 2019 issue on “Getting food on the table in cities”. Tacoli^(14)^ explains in that issue’s editorial that not only do most urban residents need to purchase most of their food, but that it is often their main expenditure.

### b. Small urbanizing cities in Bangladesh

Bangladesh is one of the most heavily and densely populated countries in the world,^(15)^ with a population of nearly 162 million and 1,240 people per square kilometre. Historically a rural and agrarian country, in recent decades Bangladesh has experienced rapid urbanization. By 2035, the majority of the country’s population is projected to be living in urban areas.^(16)^ The 2011 census data indicated that roughly 40 million people lived in Bangladesh’s smaller cities (those outside of the capital and major regional urban nodes, and with a population under 200,000).^(17)^ Yet these smaller Bangladeshi cities, where tens of millions of residents live in total, have received little to no academic or research attention. High density is not a defining feature of the urbanism of these cities. Instead they are characterized by an emerging sprawl in their porous boundaries. Very rural areas within the city’s boundaries compete for resources with the city’s core, where it is usual for day-waged labourers to reside in one-room structures with insecure tenure. More empirical work and data collection are required concerning the ways cities with small populations and possible closer ties to rural areas are urbanizing and evolving.

**Map 1:**
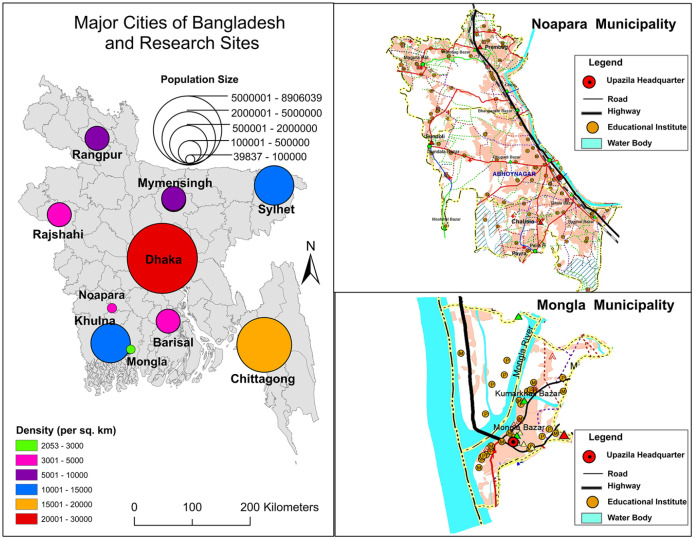
Major cities of Bangladesh and the research sites SOURCE: Juel Mahamud, ICCCAD (2020)

**Photo 1:**
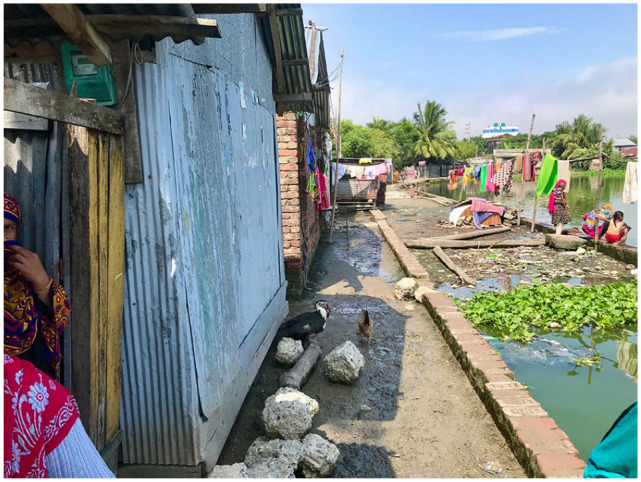
Balur Maath informal settlement in Mongla © Hanna A Ruszczyk (2019)

The two research sites for this study are Mongla and Noapara (Map 1), both located in southwestern Bangladesh. Mongla has a population of 106,000 and is adjacent to the world’s largest mangrove forest, the Sundarban, which shares a border with India. It is the home to the country’s second largest seaport and an export processing zone (EPZ) that employs over 6,000 people, the majority of whom are women. Mongla (Photo 1) has the appearance of a “sleepy city”, but is about to expand significantly in response to the central government’s expansion strategies. These include plans to double the employment capacity of the EPZ and to build an international airport nearby. Mongla struggles with water salinity and it depends on food products being transported into the city, since the immediate vicinity is unable to grow sufficient agricultural products.

Noapara is a thriving river port serving an important national transportation function. A national highway bisects the city, and the railway links Noapara to the rest of Bangladesh, as well as to India. The Bhairav River connects Noapara to the seaport of Mongla and on through the Bay of Bengal to the primary Bangladeshi port of Chittagong in southeast Bangladesh. The population of Noapara is 170,000 and its residents have a range of employment opportunities, including day labour and jobs in a well-respected jute factory. Rural-to-urban links are very evident here. Tens of thousands of people commute daily from the surrounding rural areas into Noapara for day-waged work. For more detailed information on these cities please refer to ICCCAD.^(18)^

### c. Development gains and food security in Bangladesh

Over the last three decades, despite a doubling of its population, Bangladesh has made remarkable progress from chronic food deficits in the 1970s to being self-sufficient in food, at least in terms of calorie availability.^(19)^ As poverty levels declined from 56.6 per cent in 1991/1992 to 31.5 per cent in 2010, people’s access to food also improved for all sectors of society.^(20)^ The country has also made positive progress regarding the nutritional status of the population. However, despite substantial progress in attaining food self-sufficiency at the aggregate level, a large number of people remain food insecure and hungry in the face of periodic shocks,^(21)^ and the impacts of climate change will exacerbate problems. Rice remains the main source of energy in the Bangladeshi diet, and the majority of the population consumes less than 75 percent of the daily recommended amounts for several food categories, namely eggs, pulses, milk and fruit. Owing to the impact of rapid urbanization on socioeconomic and climatic shifts, new food security concerns are emerging. Although informal settlement dwellers tend to have food security, their diet does not have the nutritional balance available to the rural population. Essential non-food costs associated with urban life (such as housing, water, fuel and schooling) restrict the ability of informal settlement dwellers to buy the recommended quantities of each food category.^(22)^

## III. Research Methodology

This research was based on interdisciplinary methods and two distinct periods of empirical work. The first fieldwork period occurred in September and October 2019 and was the foundation for the 12-month “Liveable Regional Cities in Bangladesh” project. Mongla and Noapara were selected because they present different scenarios for climate change vulnerability, and tensions between sustainable development and environmental degradation. Yet both of these academically overlooked, small, regional cities are attracting people in search of livelihood opportunities.

In this project, the 10-person international project team comprised of Bangladeshi, South African and British researchers conducted the fieldwork. There were 201 in-depth household surveys conducted (comprised of 90 questions), with an equal number of middle-class residents^(23)^ and low-income residents living in informal settlements. There were also 40 semi-structured interviews with individual residents, local and national government officials, and stakeholders such as doctors, community leaders and representatives of non-governmental organizations. In addition, four focus group discussions were conducted with residents, in each case from similar backgrounds and facing similar issues. Finally, we organized participatory theatre workshops with up to 15 residents in each city, culminating in short street theatre performances.^(24)^ These interdisciplinary methods allowed us to listen to residents and stakeholders, who detailed their lives in these small urbanizing cities, and described their aspirations and hopes for the future. This range of methods allowed us to triangulate findings and to arrive at layered understandings of tensions and areas of agreement related to the concept of liveability and everyday lived experiences amongst residents.

The second period of empirical data collection took place between the second half of May and July 2020. The purpose of this research was to learn how the COVID-19 lockdown impacted the two regional cities and some of the respondents from the Liveability project. Due to the difficult situation created by the COVID-19 lockdown, we were unable to physically travel to the cities and decided to conduct interviews by telephone. The fieldwork was subsequently disrupted by Cyclone Amphan. Ultimately, 15 formal telephone-based semi-structured interviews with key informants from the first fieldwork phase were conducted. Each interview lasted approximately one hour and was immediately transcribed from Bengali into English. Subsequently, the team of three Bangladeshi interviewers discussed each of the interviews with the two UK-based researchers. This process allowed for nuance and additional questions to emerge. There were also informal discussions between interviewers and some of the key respondents from the 2019 fieldwork period with whom we had established a good rapport. These key informants represent a cross-section of residents (from both cities, from low-income and middle-income households, men and women), stakeholders and government officials with whom we engaged with during the 2019 fieldwork.

## IV. Contextualizing the Covid-19 Lockdown

In both Mongla and Noapara, everyday life can be perceived as difficult by visitors, but residents generally like their lives and their respective cities. Both towns remain exposed to a range of natural hazards, with Mongla being particularly vulnerable to cyclones, storm surges and salinity intrusion. Indeed, Cyclone Amphan struck Bangladesh during the lockdown^(25)^ and destroyed several houses in Mongla Municipality.^(26)^ However, no fatalities were reported in either city.

The two cities enacted the lockdown at the same time as the rest of the country. Most residents in the two cities initially expected the lockdown to last no more than two weeks. Residents had to stay indoors because, according to an elderly male respondent from Mongla, if they stepped out, they risked being beaten or fined by the police, who strictly enforced the lockdown. All residents interviewed in both cities were willing to abide by the lockdown in order to preserve lives, saying *“we are all in this together”* (community leader from Noapara). The male elder said, *“the government has done the right things and took the right responses”*. All the residents respected the guidance they had been given regarding hygiene. This included washing hands, wearing masks when outside and keeping their homes cleaner than usual if possible. With some exceptions, all residents were staying at home, striving to follow the rules. Food markets were open each day for a four-hour period in the morning. Most of the shopping continued to be undertaken by male members of the household.

**Figure 1:**
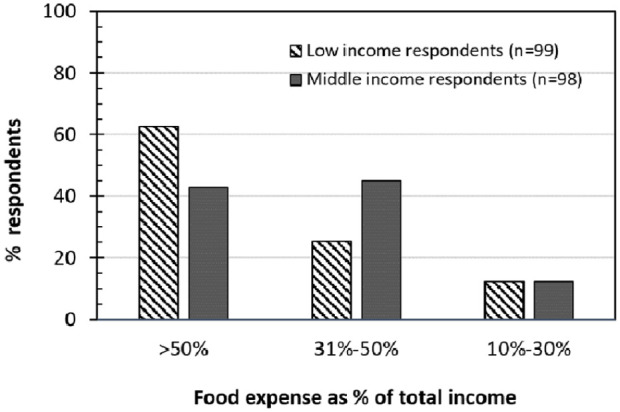
Food expense as % of total income in September 2019 NOTES: There were 201 total survey respondents; 4 respondents did not answer the specific question.

During the initial household survey in September 2019, it was apparent that buying food from the local markets was the primary means of sourcing food for residents in both Mongla and Noapara. As highlighted in Figure 1, food-related expenses were reported in both small cities to represent a major share of the total income of residents. Approximately 63 per cent of low-income respondents and 43 per cent of middle-income respondents spend over 50 per cent of their total income on food. This proportion is high compared to that found by research conducted in Vietnam,^(27)^ where 40 per cent of household expenditure of lower-income groups was on food. Research on small towns in Nepal and Cambodia suggests that the proportion of income allocated to food expenses is also significant, depending on occupation and income.^(28)^

**Figure 2:**
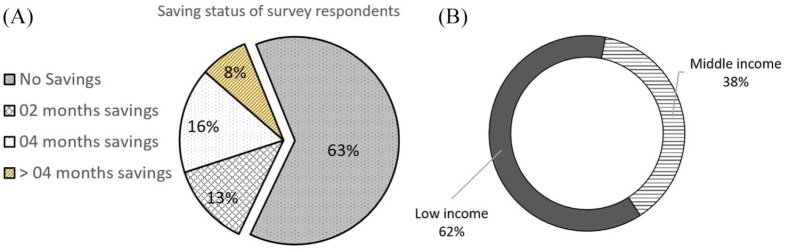
A) Saving status of survey respondents in September 2019 (n=201) B) Distribution of respondents with no savings (n=127)

While most respondents reported earning enough money to cover their ongoing expenses in 2019, most were unable to save. Data from the household survey suggest that nearly 63 per cent of the survey respondents had no savings, and the majority of this group (62 per cent) were low-income respondents (Figure 2). Apart from those reporting no savings, a further 21 per cent of respondents indicated that they had enough savings to cover two months of expenses.

## V. Key Findings Regarding Food (In)Security and Coping Mechanisms

In reporting our findings, we pay close attention first to changes in food security due to the COVID-19-induced lockdown; second, to coping strategies utilized by residents; and third, to a comparison of food security between these small cities and large cities.

### a. Food (in)security in the two cities

For both cities, food is primarily produced outside of the municipal boundaries. During the lockdown, the cost of food increased exponentially in Mongla due to its geographic location, distant from other cities, and with most people living across a river from where food supplies enter the city.

#### Changes in food consumption and diet due to the COVID-19 lockdown

In the September 2019 household survey, 84 per cent of the 201 respondents reported eating three meals daily. In the aftermath of the lockdown, most respondents from both cities reported a total, or near-total, loss of income and livelihoods. Consequently, both the quantity and quality of food being consumed by these informal settlement residents in the two cities have been significantly negatively impacted. An elderly male respondent from Mongla, for instance, stated that his family now hopes for one *“full meal every day”*. The types of food being consumed by his family during the lockdown include mashed potatoes (*alu vorta*) and rice with salt. The consumption of fish is significantly reduced due to its cost. Other respondents reported similar food choices and a primarily starchy diet. A female resident from Noapara explained:

“We are consuming less foods in quantity and quality. Now I cook less rice than before. Sometimes we can manage vegetables or spinach. As foods are dependent on our income, and I am not doing any work – so our family is dependent on the very much lower amount of my son’s earnings.”

As indicated by this woman, food consumption is directly determined by earnings. Respondents did not refer to rural linkages, nor did they refer to food staples being provided by contacts or extended family networks within rural areas. Respondents also indicated that they had reduced the proportion of nutritious food consumed, especially animal protein items such as meat, milk and poultry products, because they could not afford them. Moreover, there was a rumour^(29)^ that people could contract the virus from animal-based products. While there is no evidence to date that the virus spreads through food,^(30)^ such rumours led to a further reduction in the already low levels of consumption of nutritious food. They also affected market demand for animal-based products, especially poultry products. Overall, respondents indicated that they prioritized not being hungry over the consumption of a balanced diet.

Respondents reported that food is available in their local markets, but they pointed to two reasons for their changes in consumption: lack of affordability and, to some extent, limited access to the market. Similar changes in food consumption and diet have been reported in the region in response to disasters induced by hazards.^(31)^ Rahman et al.^(32)^ also observed similar patterns during the COVID-19 pandemic in informal settlements in larger urban areas, and in rural areas in Bangladesh.

#### Relationship between food security and household financial security

The impact of the pandemic on livelihoods has been *“a disastrous situation for people from all spheres of lif*e*”*, according to a community leader and snack shop owner in an informal settlement adjacent to the railway tracks in Noapara. Household incomes dropped markedly, and most interviewees reported a 75 to 100 per cent loss of income. Also in many cases 100 per cent of household expenditures was going towards food. Respondents had no available funds to pay for such regular and ongoing expenses as rent, utilities and other essentials. A young female respondent from Mongla expressed her situation:

“We spend all of the money to buy food and still we are unable to buy enough food items.”

The serious impact on the purchasing capacity of residents and on household food security in both cities applied even to those who are described as middle class by fellow residents or who self-describe as middle class. The important exception in terms of food security are residents who have a guaranteed income from the state or non-governmental organizations (they are often middle-class residents). Mackay^(33)^ similarly reports findings from secondary cities of Uganda on the importance of a “reliable salaried employment” on food security. The middle-class residents in Mongla and Noapara were also more likely to have savings to cover two months of expenses.

Most residents live in a financially precarious state in their everyday lives. Figure 2 explains why almost all the interviewees (especially low-income respondents) in both cities reported severe economic hardship in May–July 2020, particularly after the first month of lockdown. Informal food providers, including street vendors and vegetable/fruit peddlers (who usually go from door to door), play an essential role in the food systems of both small cities. The strict lockdown in both cities meant shutting down these businesses, which resulted not only in the loss of livelihoods for the owners, but also limited access to food for residents. A middle-class resident in Noapara explained:

“I can understand the pain of the people who are involved in food-related business or work. We are suffering a lot. We are not allowed to open shop and the market can only open for four hours a day. People can’t sell their products normally and they are facing losses, on the other hand in some cases, because of fewer customers. Vendors need to sell products at low prices.”

This quote highlights the need for awareness of the range of employment and livelihood strategies, and the importance of social protection measures, for urban informal food workers and other key microbusinesses in these small cities where the line between formal and informal work is often blurred. Those in need might not necessarily be limited to informal settlement dwellers, but can also include the rest of the working poor and lower-middle income residents in these small, sometimes overlooked cities. The quote also highlights where and how most people purchase their food in these cities – in small local shops and the municipal market.

### b. Coping strategies to deal with food (in)security

We identified several coping strategies during the COVID-19 pandemic for residents in these two small cities: storing food (at the most, enough for one month), skipping meals or curtailing their consumption, increasing the share of total expenditure allocated to food, accessing food relief or, lastly, taking loans from neighbours, friends or loan sharks during the second month of lockdown.

In both cities residents reported storing similar types of food. However, the quantity differed depending on the financial condition of the respondents. In Noapara, the community leader explained that his household had stockpiled 25 kilograms of rice and some dry goods, which allowed them to survive for one month. Some low-income respondents indicated that they did not have *“the money to stockpile”*; otherwise they would have done so. Middle-class interviewees with savings mentioned that they did not store food since it was still available in the market. They also explained that they were only purchasing what they needed in order to ensure that food staples were available for other people.

**Photo 2:**
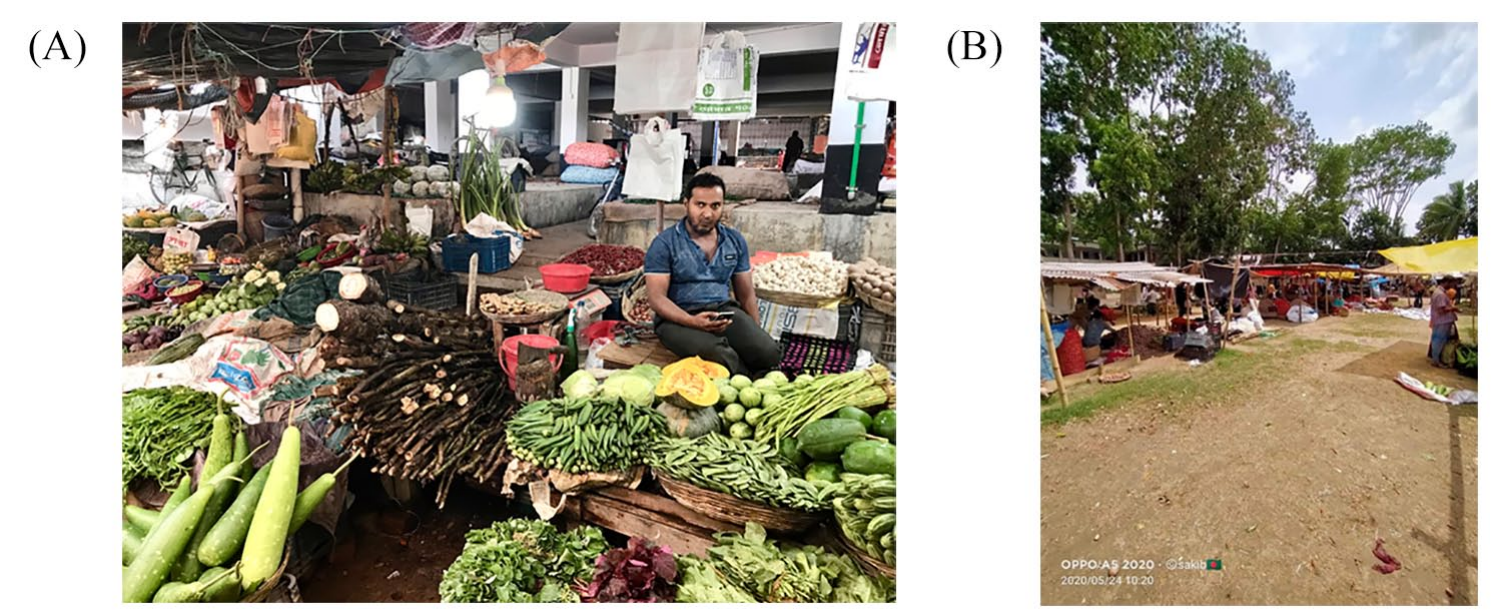
A) Noapara food market in October 2019 B) Makeshift Noapara food market in May 2020 Photo 2A © Hanna A Ruszczyk (2019) Photo 2B © Shakhawat Hossain (2020)

The local authorities have played an important role in providing food support during the lockdown and ensuring that residents could survive the crisis. First, they provided timely and immediate food relief packages in May 2020 to low-income residents in both cities. The amount of food given in Mongla was 10 kilograms of rice, 2 kilograms of potatoes, 500 grams of pulses and some soap. In Noapara, residents received 20 kilograms of rice, 1 kilogram of onions, 1 kilogram of pulses such as lentils, some soap and cooking oil. Second, in both cities, food markets with social distancing measures in place were established (see Photo 2A for the Noapara food market in October 2019 and Photo 2B for the temporary socially distanced food market in May 2020). In addition to the local authorities, non-governmental organizations, the military and the *upazila* (the second lowest tier of regional administration in Bangladesh) distributed food packages during the lockdown. It is unclear if this response was fragmented or coordinated. Although the food relief was useful, it was quite clear that it did not reach everyone in need; nor did it give adequate consideration to households maintaining a balanced diet.

Kim et al.’s study of food aid in Tanzania and Bangladesh^(34)^ suggests that it could be a wise intervention for food aid, granted primarily for food security, to target the poor suffering from food shortages. All the low-income interviewees in our study suggested that the local government’s food relief provided them support at least temporarily. What is noteworthy from interviews with (lower) middle-income households (including those with small businesses) is that they appear to be suffering as much as, if not more than, low-income households in the informal settlements because they do not qualify for food relief or social safety net programmes. A young man from Mongla explained:

“We are not very well-off and come from a middle-class family. We have no income and we cannot even ask for money from others.”

This may lead to hidden or invisible food insecurity among middle-income households. Some households (especially those with more than one member earning an income) in the informal settlements also expressed their dismay and humiliation at needing to access food relief for the first time in their lives. The feeling of shame was evident in interviews among people who were accustomed to offering help rather than asking for it.

Due to the protracted length and hardship of the unprecedented crisis of COVID-19 and the subsequent loss of income sources, the majority of residents appear to have taken out loans to buy food. Borrowing for food expenses has also been documented as a coping strategy in the aftermath of floods, droughts and cyclones.^(35)^ The 2019 household survey pointed to four sources of loans to cover everyday needs: friends and family (which had been used by 35 per cent of respondents), community (22 per cent), NGOs (18 per cent), and other sources, e.g. pawnbrokers, loan sharks (25 per cent). During the pandemic, interviewees also reported taking loans from their friends and neighbours, reflecting the importance of social capital to cope with disasters. However, several interviewees stated that their traditional support systems could not be accessed because everyone was going through the same hardship and had no money to spare.

### c. Comparing food security in small cities and large cities

During this study, a set of broad similarities and differences in food security between settlements in small cities such as Mongla and Noapara and large cities such as Dhaka were identified.

#### Similarities

Our COVID-19 lockdown findings from the two small cities (high food insecurity among the low-income households in Mongla and Noapara and associated coping strategies) appear to be similar to those reported by studies in informal settlements in the Bangladeshi capital, the mega-city Dhaka.^(36)^ Informal settlement dwellers in big cities also reported curtailing food consumption, resorting to cheap starchy staples, using savings (wherever possible), borrowing, or relying on grocery store credit and food relief to cope with food insecurity. The cost of essential items rose during the lockdown in small cities as well as in large cities. Average food prices in Dhaka were reported to be 20 per cent above pre-lockdown prices on 19 April 2020.^(37)^ In Mongla, there were increases in the price of food during the month of May, according to the respondents. In Noapara, there were no price increases in May; this may be due to the strategic transportation and distribution location of Noapara. These insights are based on information provided in interviews during May–June 2020. In both cities, the price of food had increased by July (four months after lockdown was introduced).

The importance of social capital and the lack of coverage of formal social safety nets were also apparent in both types of cities.^(38)^ It was quite clear that the crisis of hunger and food insecurity preceded the crisis of the pandemic. This pandemic essentially exposed the existing inequalities that are present in large and small cities in Bangladesh alike.^(39)^

#### Differences

Despite the similarities, there are also distinct differences between the two types of cities related to how residents procured food. Residents in Mongla and Noapara primarily relied on their local markets and neighbourhood grocery stores for procuring their food. Large cities such as Dhaka on the other hand possess greater diversity of food outlets (e.g. types of local markets, supermarkets, street food, fast food, restaurants, and relief providers). In large cities, retail competition for food items and cheaper options for purchasing food are more prevalent than in smaller cities.

Compared to mega-cities such as Dhaka, smaller cities tend to have greater opportunities for practising urban agriculture. Some small cities close to rural areas may also have better food security. During a shutdown of transport services, local food producers may also find it easier to sell their products to nearby smaller city markets than to their original large city destinations.

While negative coping strategies such as distress sales of assets were reported in Dhaka,^(40)^ this did not appear to be a major coping strategy in other places in Bangladesh.^(41)^ Nor did respondents in our study mention distress sales. Taylor^(42)^ documents that in Dhaka, survival strategies among some informal settlement dwellers included *“changing jobs to be mobile food vendors (permitted because they are considered essential services), or breaking lockdown rules, going out as rogue rickshaw drivers”*. However, such strategies were not reported in our study, probably due to the fact that the lockdown was strictly enforced in both cities. Residents were threatened with devastating fines or beatings.

Finally, there is less distance between the local government and residents in smaller cities, both physically and mentally. While local government officials in small cities may have limited capacity and financial resources, the greater proximity enables local governments in these cities to act promptly and decisively. This was evident in the temporary opening of the local food market in each city that allowed for social distancing measures. Significant trust in the local government in the two case study cities also probably played a role in this regard. However, these aspects need to be further researched.

## VI. Concluding Thoughts

The pandemic is an evolving urban crisis that will likely have cascading and lasting consequences on cities and their residents for decades to come. Based on an empirical analysis of the situation in Mongla and Noapara in Bangladesh, and a comparison of the experiences of informal settlement dwellers and middle-class residents, this paper provides a nuanced understanding of food (in)security in small urbanizing cities of the global South. This research shows that COVID-19 is disproportionally impacting low-income households who live, in any case, in everyday tenuous circumstances. However, it is also having an impact on middle-class households. Under the added pressure of the pandemic, residents have decreased the quantity and quality of food they consume, responding to severe economic hardship related to their almost complete loss of income. Their coping strategies include storing food, curtailing consumption, increasing the share of total expenditure allocated to food, accessing food relief and taking out loans. The nine-week COVID-19 lockdown has not only affected those who have been historically marginalized (informal settlement dwellers), but is also threatening the invisible fabric of small cities, the shopkeepers, and the microbusinesses that may be formal or informal in their legal status. There are both similarities and differences between small cities and large cities in Bangladesh in their experiences of the COVID-19 lockdown’s impact on food security.

There will be more lessons to learn from the lockdown; it is still early in the evolving reality of the pandemic. This research paper highlights the vulnerability of a multiplicity of poor people in small cities who are often not covered by social safety net programmes in Bangladesh. This paper opened with the United Nations declaration about the pandemic’s reach going beyond health to undermine achieved development gains. This is important to remember when we think about the evolving impact of COVID-19 on food security in urbanizing areas. To create socially inclusive, economically equitable and environmentally sustainable development requires closer attention to where people live in the world – including small urbanizing cities where most residents are struggling, trying to make do through their own efforts. There is a need for careful attention to be given to international, national and local strategies to support people when they rebuild their lives, and to create safety nets appropriate for urban households and communities in different settings.

## Footnotes

1. UNDP (2020), *COVID-19 and Human Development: Assessing the Crisis, Envisioning the Recovery*, United Nations Development Programme, New York, available at http://hdr.undp.org/en/hdp-covid.

2. Wilkinson, Annie (2020), “Local response in health emergencies: key considerations for addressing the COVID-19 pandemic in informal urban settlements”, *Environment and Urbanization* Vol 32, No 2, pages 503–522, available at https://journals.sagepub.com/doi/full/10.1177/0956247820922843.

3. DGHS (2020), *Corona COVID-19 Virus Dashboard 2020*, Directorate General of Health Services, available at http://103.247.238.81/webportal/pages/covid19.php.

4. ADB (2020), *The Economic Impact of the COVID-19 Outbreak on Developing Asia*, Asian Development Bank, available at https://www.adb.org/sites/default/files/publication/571536/adb-brief-128-economic-impact-covid19-developing-asia.pdf.

5. Arkom Indonesia (2020), *COVID-19 Pandemic – Emergency Response – Arkom Indonesia*, concept note, University College London–Development Planning Unit (UCL–DPU), Knowledge in Action for Urban Equality (KNOW), available at https://45279888-944b-4def-99e5-22ea75128921.filesusr.com/ugd/623440_93cbd7b0c0074f289be7dec1d99f763c.pdf; also Cooper-Knock, S J, F Anciano, M Dube, M Majola and B M Papane (2020), *Lockdown Diaries: 70 Participants across Cape Town Share Their Experiences of the Covid-19 Lockdown*, available at https://lockdowndiaries.org; Huq, S (2020), “Tales of local resilience on the frontline of COVID and climate change”, blog post, 14 July, International Center for Climate Change and Development, available at http://www.icccad.net/dr-saleemul-huq-media/tales-of-local-resilience-on-the-frontlines-of-covid-and-climate-change; Khan, M (2020), “Dhaka, Bangladesh: how one woman’s idea unleashed the power of community spirit”, blog post, 13 July, International Center for Climate Change and Development, available at http://www.icccad.net/voices-from-the-frontline/power-of-community-spirit; and UCL-DPU and Arkom Indonesia (2020), *Viewing Urban Informal Community Homes Conditions & Support in the COVID-19 Pandemic Period*, illustrative guide, University College London–Development Planning Unit, Knowledge in Action for Urban Equality (KNOW), available at https://45279888-944b-4def-99e5-22ea75128921.filesusr.com/ugd/623440_a808c390d28640e5975dd6180c501e43.pdf.

6. Rahman, H Z, N Das, I Matin, M A Wazed, S Ahmed, N Jahan and U Zillur (2020), *Livelihoods, Coping and Support During Covid-19 Crisis*, PPRC-BIGD response research, Power and Participation Research Centre and BRAC Institute of Governance and Development, available at https://bigd.bracu.ac.bd/wp-content/uploads/2020/05/PPRC-BIGD-Final-April-Survey-Report.pdf.

7. Ali, K A and M Rieker (2008), “Introduction: urban margins”, *Social Text* Vol 26, No 2, pages 1–12, page 2, available at https://read.dukeupress.edu/social-text/article/26/2%20(95)/1/33558/IntroductionUrban-Margins.

8. Hardoy, J E and D Satterthwaite (1989), *Squatter Citizen: Life in the Urban Third World*, Earthscan, London.

9. Satterthwaite, D (2017), “The impact of urban development on risk in sub-Saharan Africa’s cities with a focus on small and intermediate urban centres”, *International Journal of Disaster Risk Reduction* Vol 26, pages 16–23, available at http://dx.doi.org/10.1016/j.ijdrr.2017.09.025; also Marais, L, E Nel and R Donaldson (editors) (2016), *Secondary Cities and Development*, Routledge, London.

10. Ruszczyk, H A, E Nugraha and I de Villiers (2020) (editors), *Overlooked Cities: Power, Politics and Knowledge Beyond the Urban South*, Routledge; also Price, M and H A Ruszczyk (2019), “On what basis are urban futures being decided?”, Geography Directions blog, 12 August, available at https://blog.geographydirections.com/2019/06/28/on-what-basis-are-urban-futures-being-decided.

11. Simone, A M (2014), “The missing people: an urban majority”, in S Parnell and S Oldfield (editors), *The Routledge Handbook on Cities of the Global South*, 1st edition, Routledge, London, pages 322–336, page 322.

12. Birkmann, J, T Welle, W Solecki, S Lwasa and M Garschagen (2016), “Boost resilience of small and mid-sized cities”, *Nature* Vol 537, pages 605–608, available at http://dx.doi.org/10.1038/537605a.

13. Mackay, H (2019), “Food sources and access strategies in Ugandan secondary cities: an intersectional analysis”, *Environment and Urbanization* Vol 31, No 2, pages 375–396.

14. Tacoli, C (2019), “Editorial: The urbanization of food insecurity and malnutrition”, *Environment and Urbanization* Vol 31, No 2, pages 371–374.

15. World Bank (n.d.), *Population Density (People per Sq. Km of Land Area) - Bangladesh*, available at https://data.worldbank.org/indicator/EN.POP.DNST?locations=BD.

16. UNDP (2019), “Why a national urban policy should be our top priority”, blog post, 31 October, United Nations Development Programme, available at https://www.bd.undp.org/content/bangladesh/en/home/blog/2019/october/31/why-a-national-urban-policy-should-be-our-top-priority.html.

17. UNFPA (2016), *Urbanization and Migration in Bangladesh*, Paper No 176, United Nations Population Fund, available at https://bangladesh.unfpa.org/en/publications/urbanization-and-migration-bangladesh.

18. ICCCAD (2020a), *Mongla Dissemination Brief*, “Liveable Regional Cities in Bangladesh” project, International Centre for Climate Change and Development, available at http://www.icccad.net/wp-content/uploads/2020/06/Mongla-Dissemination-Brief-June-2020.pdf; also ICCCAD (2020b), *Noapara Dissemination Brief*, “Liveable Regional Cities in Bangladesh” project, International Centre for Climate Change and Development, available at http://www.icccad.net/wp-content/uploads/2020/06/Noapara-Dissemination-Brief-June-2020.pdf.

19. Osmani, S R, T Ahmed, N Hossain and S Huq (2016), *Strategic Review of Food Security and Nutrition in Bangladesh*, World Food Programme.

20. See reference 19.

21. See reference 19.

22. Satterthwaite, D (2004), *The Under-Estimation of Urban Poverty in Low and Middle-Income Nations, *Working Paper 14, International Institute for Environment and Development, available at https://pubs.iied.org/pdfs/9322IIED.pdf; also Satterthwaite, D, G McGranahan and C Tacoli (2010), “Urbanization and its implications for food and farming”, *Philosophical Transactions of the Royal Society B: Biological Sciences* Vol 365, No 1554, pages 2809–2820.

23. The middle-class residents were identified by the surveyors asking random, varied local residents, “Where do the middle-class people live?” According to the local respondents, lower-income people also live in areas where middle-class people reside. Education was repeatedly stressed as a signifier of middle-class identity. In a number of cases, elders or family heads were not educated, but the second generation had completed higher education or was currently attending school. The middle-class residents were both homeowners and tenants.

24. Access to the outputs (two films, surveys, photography exhibit, two blogs and two dissemination briefs) from the first fieldwork period can be found at ICCCAD (2020c), *Liveable Regional Cities in Bangladesh*, available at http://www.icccad.net/programmes/resilient-livelihood/liveable-regional-cities-in-bangladesh and https://www.dur.ac.uk/ihrr/research/themes/disruptedcities/liveable.cities.

25. Gettleman, J and S Yasir (2020), “Cyclone Amphan’s death toll rises to 80 in India and Bangladesh”, *New York Times*, 21 May 2020, available at https://www.nytimes.com/2020/05/21/world/asia/cyclone-amphan-india-bangladesh.html.

26. *BBC News* (2020), video interview with a resident of Mongla Port Municipality affected by Cyclone Amphan, 21 May, available at https://www.bbc.com/bengali/live/news-52737422.

27. Wertheim-Heck, S, J E Raneri and P Oosterveer (2019), “Food safety and nutrition for low-income urbanites: exploring a social justice dilemma in consumption policy”, *Environment and Urbanization* Vol 31, No 2, pages 397–420.

28. Boonyabancha, S, T Kerr, L Joshi and C Tacoli (2019), “How the urban poor define and measure food security in Cambodia and Nepal”, *Environment and Urbanization* Vol 31, No 1, pages 517–532.

29. During the early days of the pandemic a rumour spread, further fuelled through social media, suggesting that coronavirus might be transmitted through foods such as poultry products, fish and meat. This rumour was not specific to the two cities, but was spreading across the nation as well as in other countries in South Asia. Consumption of these food items decreased during the lockdown for this reason as well.

30. CDC (2020), *Food and Coronavirus Disease 19 (COVID-19)*, https://www.cdc.gov/coronavirus/2019-ncov/daily-life-coping/food-and-COVID-19.html.

31. Béné, C, J Waid, M Jackson-deGraffenried, A Begum, M Chowdhury, V Skarin, A Rahman, N Islam, N Mamnun, K Mainuddin, K and S M A Amin (2015), *Impact of Climate-Related Shocks and Stresses on Nutrition and Food Security in Selected Areas of Rural Bangladesh*, World Food Programme, Dhaka, 150 pages; also Paul, S K and J K Routray (2011), “Household response to cyclone and induced surge in coastal Bangladesh: coping strategies and explanatory variables”, *Natural Hazards* Vol 57, No 2, pages 477–499, available at http://dx.doi.org/10.1007/s11069-010-9631-5.

32. See reference 6.

33. See reference 13, page 385.

34. Kim, Y, H Sohn and B Park (2019), “Make the village better: An evaluation of the Saemaul Zero Hunger Communities Project in Tanzania and Bangladesh”, *World Development* Vol 124, available at https://doi.org/10.1016/j.worlddev.2019.104652.

35. Béné, C, P Oosterveer, L Lamotte, I D Brouwer, S de Haan, S D Prager E F Talsma and C K Khoury (2019), “When food systems meet sustainability – current narratives and implications for action”, *World Development* Vol 113, pages 116–130, available at http://dx.doi.org/10.1016/j.worlddev.2018.08.011.

36. See reference 6; also Rashid, S F, B Aktar, N Farnaz, S Theobald, S Ali, W Alam and K Ozano (2020), “Fault-lines in the public health approach to covid-19: recognizing inequities and ground realities of poor residents lives in the slums of Dhaka City, Bangladesh”, *Social Sciences & Humanities Open*, available at https://ssrn.com/abstract=3608577; and Taylor, J (2020), “How Dhaka’s urban poor are dealing with COVID-19”, *Urban Matters*, 1 July, International Institute for Environment and Development, available at https://www.iied.org/how-dhakas-urban-poor-are-dealing-covid-19.

37. FAO (2020), *Impact of COVID-19 on Food Security and Urban Poverty*, Food and Agriculture Organization Situation Report 6, 2–8 May 2020.

38. See reference 6; also see reference 36, Taylor (2020); and Rahman, M F and H A Ruszczyk (2020), “Coronavirus: how lockdown exposed food insecurity in a small Bangladeshi city”, *The Conversation*, 16 July, available at https://theconversation.com/coronavirus-how-lockdown-exposed-food-insecurity-in-a-small-bangladeshi-city-140684.

39. See reference 36, Rashid et al. (2020); also see reference 38, Rahman and Ruszczyk (2020).

40. See reference 36, Taylor (2020).

41. See reference 6.

42. See reference 36, Taylor (2020).

## References

[bibr1-0956247820965156] ADB (2020), The Economic Impact of the COVID-19 Outbreak on Developing Asia, Asian Development Bank, available at https://www.adb.org/sites/default/files/publication/571536/adb-brief-128-economic-impact-covid19-developing-asia.pdf.

[bibr2-0956247820965156] Ali K A Rieker M (2008), “Introduction: urban margins”, Social Text Vol 26, No 2, pages 1–12, available at https://read.dukeupress.edu/social-text/article/26/2%20(95)/1/33558/IntroductionUrban-Margins.

[bibr3-0956247820965156] Arkom Indonesia (2020), COVID-19 Pandemic – Emergency Response – Arkom Indonesia, concept note, University College London–Development Planning Unit (UCL–DPU), Knowledge in Action for Urban Equality (KNOW), available at https://45279888-944b-4def-99e5-22ea75128921.filesusr.com/ugd/623440_93cbd7b0c0074f289be7dec1d99f763c.pdf.

[bibr4-0956247820965156] *BBC News* (2020), video interview with a resident of Mongla Port Municipality affected by Cyclone Amphan, 21 May, available at https://www.bbc.com/bengali/live/news-52737422.

[bibr5-0956247820965156] Béné C Oosterveer P Lamotte L Brouwer I D de Haan S Prager S D Talsma E F Khoury C K (2019), “When food systems meet sustainability – current narratives and implications for action”, World Development Vol 113, pages 116–130, available at 10.1016/j.worlddev.2018.08.011.

[bibr6-0956247820965156] Béné C Waid J Jackson-deGraffenried M Begum A Chowdhury M Skarin V Rahman A Islam N Mamnun N Mainuddin K Amin S M A (2015), Impact of Climate-Related Shocks and Stresses on Nutrition and Food Security in Selected Areas of Rural Bangladesh, World Food Programme, Dhaka, 150 pages.

[bibr7-0956247820965156] Birkmann J Welle T Solecki W Lwasa S Garschagen M (2016), “Boost resilience of small and mid-sized cities”, Nature Vol 537, pages 605–608, available at 10.1038/537605a. 27680922

[bibr8-0956247820965156] Boonyabancha S Kerr T Joshi L Tacoli C (2019), “How the urban poor define and measure food security in Cambodia and Nepal”, Environment and Urbanization Vol 31, No 1, pages 517–532.

[bibr9-0956247820965156] CDC (2020), Food and Coronavirus Disease 19 (COVID-19), https://www.cdc.gov/coronavirus/2019-ncov/daily-life-coping/food-and-COVID-19.html.

[bibr10-0956247820965156] Cooper-Knock S J Anciano F Dube M Majola M Papane B M (2020), Lockdown Diaries: 70 Participants across Cape Town Share Their Experiences of the Covid-19 Lockdown, available at https://lockdowndiaries.org.

[bibr11-0956247820965156] DGHS (2020), Corona COVID-19 Virus Dashboard 2020, Directorate General of Health Services, available at http://103.247.238.81/webportal/pages/covid19.php.

[bibr12-0956247820965156] FAO (2020), Impact of COVID-19 on Food Security and Urban Poverty, Food and Agriculture Organization Situation Report 6, 2–8 May 2020.

[bibr13-0956247820965156] Gettleman J Yasir S (2020), “Cyclone Amphan’s death toll rises to 80 in India and Bangladesh”, New York Times, 21 May 2020, available at https://www.nytimes.com/2020/05/21/world/asia/cyclone-amphan-india-bangladesh.html.

[bibr14-0956247820965156] Hardoy J E Satterthwaite D (1989), Squatter Citizen: Life in the Urban Third World, Earthscan, London.

[bibr15-0956247820965156] Huq S (2020), “Tales of local resilience on the frontline of COVID and climate change”, blog post, 14 July, International Center for Climate Change and Development, available at http://www.icccad.net/dr-saleemul-huq-media/tales-of-local-resilience-on-the-frontlines-of-covid-and-climate-change.

[bibr16-0956247820965156] ICCCAD (2020a), Mongla Dissemination Brief, “Liveable Regional Cities in Bangladesh” project, International Centre for Climate Change and Development, available at http://www.icccad.net/wp-content/uploads/2020/06/Mongla-Dissemination-Brief-June-2020.pdf.

[bibr17-0956247820965156] ICCCAD (2020b), Noapara Dissemination Brief, “Liveable Regional Cities in Bangladesh” project, International Centre for Climate Change and Development, available at http://www.icccad.net/wp-content/uploads/2020/06/Noapara-Dissemination-Brief-June-2020.pdf.

[bibr18-0956247820965156] ICCCAD (2020c), Liveable Regional Cities in Bangladesh, available at http://www.icccad.net/programmes/resilient-livelihood/liveable-regional-cities-in-bangladesh and https://www.dur.ac.uk/ihrr/research/themes/disruptedcities/liveable.cities.

[bibr19-0956247820965156] Khan M (2020), “Dhaka, Bangladesh: how one woman’s idea unleashed the power of community spirit”, blog post, 13 July, International Center for Climate Change and Development, available at http://www.icccad.net/voices-from-the-frontline/power-of-community-spirit.

[bibr20-0956247820965156] Kim Y Sohn H Park B (2019), “Make the village better: An evaluation of the Saemaul Zero Hunger Communities Project in Tanzania and Bangladesh”, World Development Vol 124, available at 10.1016/j.worlddev.2019.104652.

[bibr21-0956247820965156] Mackay H (2019), “Food sources and access strategies in Ugandan secondary cities: an intersectional analysis”, Environment and Urbanization Vol 31, No 2, pages 375–396.

[bibr22-0956247820965156] Marais L Nel E Donaldson R (2016), Secondary Cities and Development, Routledge, London.

[bibr23-0956247820965156] Osmani S R Ahmed T Hossain N Huq S (2016), Strategic Review of Food Security and Nutrition in Bangladesh, World Food Programme.

[bibr24-0956247820965156] Paul S K Routray J K (2011), “Household response to cyclone and induced surge in coastal Bangladesh: coping strategies and explanatory variables”, Natural Hazards Vol 57, No 2, pages 477–499, available at 10.1007/s11069-010-9631-5.

[bibr25-0956247820965156] Price M Ruszczyk H A (2019), “On what basis are urban futures being decided?”, Geography Directions blog, 12 August, available at https://blog.geographydirections.com/2019/06/28/on-what-basis-are-urban-futures-being-decided.

[bibr26-0956247820965156] Rahman H Z Das N Matin I Wazed M A Ahmed S Jahan N Zillur U (2020), Livelihoods, Coping and Support During Covid-19 Crisis, PPRC-BIGD response research, Power and Participation Research Centre and BRAC Institute of Governance and Development, available at https://bigd.bracu.ac.bd/wp-content/uploads/2020/05/PPRC-BIGD-Final-April-Survey-Report.pdf.

[bibr27-0956247820965156] Rahman M F Ruszczyk H A (2020), “Coronavirus: how lockdown exposed food insecurity in a small Bangladeshi city”, The Conversation, 16 July, available at https://theconversation.com/coronavirus-how-lockdown-exposed-food-insecurity-in-a-small-bangladeshi-city-140684.

[bibr28-0956247820965156] Rashid S F Aktar B Farnaz N Theobald S Ali S Alam W Ozano K (2020), “Fault-lines in the public health approach to covid-19: recognizing inequities and ground realities of poor residents lives in the slums of Dhaka City, Bangladesh”, Social Sciences & Humanities Open, available at https://ssrn.com/abstract=3608577.

[bibr29-0956247820965156] Ruszczyk H A Nugraha E de Villiers I (2020) (editors), Overlooked Cities: Power, Politics and Knowledge Beyond the Urban South, Routledge.

[bibr30-0956247820965156] Satterthwaite D (2004), The Under-Estimation of Urban Poverty in Low and Middle-Income Nations, Working Paper 14, International Institute for Environment and Development, available at https://pubs.iied.org/pdfs/9322IIED.pdf.

[bibr31-0956247820965156] Satterthwaite D (2017), “The impact of urban development on risk in sub-Saharan Africa’s cities with a focus on small and intermediate urban centres”, International Journal of Disaster Risk Reduction Vol 26, pages 16–23, available at 10.1016/j.ijdrr.2017.09.025.

[bibr32-0956247820965156] Satterthwaite D McGranahan G Tacoli C (2010), “Urbanization and its implications for food and farming”, Philosophical Transactions of the Royal Society B: Biological Sciences Vol 365, No 1554, pages 2809–2820. 10.1098/rstb.2010.0136 PMC2935117 20713386

[bibr33-0956247820965156] Simone A M (2014), “The missing people: an urban majority”, in Parnell S Oldfield S (editors), The Routledge Handbook on Cities of the Global South, 1st edition, Routledge, London, pages 322–336.

[bibr34-0956247820965156] Tacoli C (2019), “Editorial: The urbanization of food insecurity and malnutrition”, Environment and Urbanization Vol 31, No 2, pages 371–374.

[bibr35-0956247820965156] Taylor J (2020), “How Dhaka’s urban poor are dealing with COVID-19”, Urban Matters, 1 July, International Institute for Environment and Development, available at https://www.iied.org/how-dhakas-urban-poor-are-dealing-covid-19.

[bibr36-0956247820965156] UCL-DPU and Arkom Indonesia (2020), Viewing Urban Informal Community Homes Conditions & Support in the COVID-19 Pandemic Period, illustrative guide, University College London–Development Planning Unit, Knowledge in Action for Urban Equality (KNOW), available at https://45279888-944b-4def-99e5-22ea75128921.filesusr.com/ugd/623440_a808c390d28640e5975dd6180c501e43.pdf.

[bibr37-0956247820965156] UNDP (2019), “Why a national urban policy should be our top priority”, blog post, 31 October, United Nations Development Programme, available at https://www.bd.undp.org/content/bangladesh/en/home/blog/2019/october/31/why-a-national-urban-policy-should-be-our-top-priority.html.

[bibr38-0956247820965156] UNDP (2020), COVID-19 and Human Development: Assessing the Crisis, Envisioning the Recovery, United Nations Development Programme, New York, available at http://hdr.undp.org/en/hdp-covid.

[bibr39-0956247820965156] UNFPA (2016), Urbanization and Migration in Bangladesh, Paper No 176, United Nations Population Fund, available at https://bangladesh.unfpa.org/en/publications/urbanization-and-migration-bangladesh.

[bibr40-0956247820965156] Wertheim-Heck S Raneri J E Oosterveer P (2019), “Food safety and nutrition for low-income urbanites: exploring a social justice dilemma in consumption policy”, Environment and Urbanization Vol 31, No 2, pages 397–420. 10.1177/0956247819858019 PMC7340485 32704235

[bibr41-0956247820965156] Wilkinson Annie (2020), “Local response in health emergencies: key considerations for addressing the COVID-19 pandemic in informal urban settlements”, Environment and Urbanization Vol 32, No 2, pages 503–522, available at https://journals.sagepub.com/doi/full/10.1177/0956247820922843. 10.1177/0956247820922843 PMC7613852 36438604

[bibr42-0956247820965156] World Bank (n.d.), Population Density (People per Sq. Km of Land Area) - Bangladesh, available at https://data.worldbank.org/indicator/EN.POP.DNST?locations=BD.

